# Associations between food insecurity and mental health outcomes among Hispanic/Latino adolescents in Los Angeles and Miami

**DOI:** 10.1007/s00127-026-03170-9

**Published:** 2026-07-14

**Authors:** Gabriel Luna, Ryan Lee, Michelle M. Nuño, Daniel W. Soto, Seth J. Schwartz, Jennifer B. Unger

**Affiliations:** 1 Department of Population and Public Health Sciences, University of Southern California, Los Angeles, CA USA; 2 Department of Kinesiology and Health Education, University of Texas at Austin, Austin, TX USA

**Keywords:** Food insecurity, Adolescent mental health, Hispanic/Latino youth, Generalized anxiety, Depressive symptoms

## Abstract

**Purpose:**

To examine associations between household food insecurity and psychosocial outcomes, including depression, anxiety, stress, and aggression among Hispanic/Latino (HL) adolescents in two urban settings.

**Methods:**

This study surveyed HL adolescents from Los Angeles and Miami (*N* = 300) to examine associations between food insecurity and depression, anxiety, stress, and aggression. Food insecurity was dichotomized and analyzed in separate logistic or linear regression models for each outcome. Multivariable models were adjusted for site, gender, nativity, socioeconomic status, and age.

**Results:**

Food insecurity was significantly associated with greater odds of moderate/severe anxiety and depressive symptoms, even after adjusting for covariates (OR = 4.60, 95% CI: 2.51–8.51 and OR = 3.17, 95% CI: 1.77–5.77, respectively). Food insecurity was also associated with higher adolescent stress (Estimated difference in means = 0.39, 95% CI: 0.12–0.65). No significant interactions were observed between food insecurity and site. Additionally, boys reported lower stress and depressive symptoms than girls across models.

**Conclusions:**

Food insecurity is significantly associated with elevated anxiety, depression symptoms, and stress among HL adolescents. The strength of these associations highlights the serious mental health risks faced by HL youth experiencing food insecurity. These findings further underscore the need for addressing food insecurity hardships to support adolescent mental and behavioral health in HL communities.

## Introduction

Food insecurity is a chronic and significant public health issue that affects millions of people across the United States. The United Nations Food and Agriculture Organization defines food insecurity as the lack of regular access to nutritious food for normal growth, development, and an active and healthy lifestyle [1]. According to the United States Department of Agriculture Economic Research Service, 18 million in the United States (13.5% of all U.S. households), experienced food insecurity at some point during 2023, an increase from 12.8% in 2022 [2]. Persistent food insecurity has been linked to negative developmental outcomes that often result in increased levels of stress and anxiety, which may negatively impact children physiologically, psychologically, and socially [3, 4]. The effects of food insecurity span a range of diseases, conditions, and negative mental health outcomes, including iron deficiency, cardiovascular disease, cancers, asthma, sleep disturbances, depression, and suicidal ideation, among others [5–9].

Individuals from Hispanic/Latino (hereafter, HL) backgrounds, especially first- and second-generation immigrants, are disproportionately affected by food insecurity in the United States compared with non-Hispanic White households [10, 11]. In 2023, 21.9% of HL households experienced food insecurity, compared with 9.9% of non-Hispanic White households [2]. Several overlapping factors may contribute to this disparity. At the household level, food insecurity is closely tied to economic hardship and limited material resources, including poverty and unstable employment. Among immigrant and mixed-status HL families, these risks may be compounded by language barriers, acculturative stress, eligibility restrictions for public benefits, and concerns about accessing food assistance or other public programs [5, 12]. More recently, heightened immigration enforcement and anti-immigration rhetoric may further deter some families from seeking available food and social services, thereby intensifying existing vulnerabilities to food insecurity [12–14]. These food-related and structural challenges may occur during a developmental period in which mental health concerns are already common. In 2023, 39.7% of U.S. high school students reported persistent feelings of sadness or hopelessness, 28.5% reported poor mental health, 20.4% seriously considered attempting suicide, and 9.5% attempted suicide during the past year [15]. Hispanic students also reported substantial mental health burden, including persistent feelings of sadness or hopelessness and suicide attempts [15]. Food insecurity may further exacerbate these vulnerabilities by increasing chronic stress, disrupting daily routines, and intensifying family economic strain, all of which may contribute to anxiety, depressive symptoms, stress, and behavioral difficulties among adolescents. Compounding these challenges, HL adolescents often face structural barriers, such as limited availability of behavioral health services offered in adolescents’ or families’ preferred language, limited health insurance coverage for mental health care, transportation challenges, and stigma surrounding mental health, all of which may delay or prevent treatment for stress, anxiety, and depressive symptoms [16, 17]. These intersecting structural and psychosocial challenges highlight the urgent need to examine how food insecurity impacts the mental and behavioral health of HL youth.

To contextualize the current study, it is important to consider local demographic and economic factors that may influence food insecurity and adolescent health. Miami-Dade and Los Angeles counties differ from national averages in several key demographic and economic categories relevant to food insecurity and adolescent health. Both counties have substantially higher percentages of HL residents (69.1% in Miami-Dade and 48.6% in Los Angeles) than the national average of 19.5% [18]. Additionally, both counties reflect higher levels of household crowding, a common proxy for low socioeconomic status [19], with an average of 2.74 persons per household in Miami-Dade and 2.85 in Los Angeles, compared to the national average of 2.54 [18]. Economic data further illustrate disparities in these two large urban areas, as both regions experience persistent poverty above the national level of 11.1%, with Miami-Dade reporting 13.7% and Los Angeles reporting 14.1% below the federal poverty line [18]. These local conditions situate the present study within two urban counties where HL adolescents and families may be exposed to overlapping forms of economic strain, household resource constraints, and barriers to health and social services.

Although studies have examined food insecurity among HL youth, comparatively little work has examined associations between household food insecurity and multiple psychosocial outcomes among HL adolescents across distinct urban HL contexts [20, 21]. Moreover, little is known about how food insecurity relates to psychosocial functioning across diverse urban settings. Accordingly, the present study examined the relationship between household food insecurity and psychosocial outcomes, including anxiety, depressive symptoms, stress, and aggression, among HL adolescents living in Los Angeles and Miami-Dade counties. Our study contributes to a growing body of literature on how structural vulnerabilities shape psychosocial health in HL adolescents. We hypothesized that household food insecurity would be associated with higher levels of anxiety, depressive symptoms, stress, and aggression among HL youth. Given the limited evidence for site-specific differences in these associations, our analyses of food insecurity by site interactions were exploratory.

## Methods

### Procedures

Data were drawn from a larger multi-phase mixed methods study described elsewhere [22, 23]. The present analyses used baseline survey data collected in the Spring and Summer of 2020. Adolescent ages ranged from 14 to 17 years old. Participants attended one of three high schools, including two from Los Angeles County and one high school in Miami-Dade County. Eligibility criteria included self-identifying as Hispanic/Latino, either being born or having at least one parent who was born in a Spanish-speaking country, and being fluent in English and/or Spanish. Parents and adolescents were recruited electronically through phone calls, text messages, and emails. Due to the COVID-19 pandemic, study information, parental consent, youth assent, and survey administration were completed virtually. After electronic consent and youth assent were obtained, participants completed the survey through REDCap or Qualtrics. Participants received a $15 gift card for participation. Study procedures were reviewed and approved by the Institutional Review Boards at the University of Southern California and the University of Miami.

### Measures

#### Food Insecurity

Food insecurity was assessed using the 9-item United States Department of Agriculture (USDA) Economic Research Service (ERS) Household Food Security Module for youth [24, 25]. Each item captures an aspect of food-related hardship experienced in the last year (e.g., skipping meals, going hungry, or not eating for a whole day due to lack of resources). Summed scores were used to generate a total food insecurity score ranging from 0 to 9, with higher scores indicating greater food insecurity. Following USDA guidelines, participants who scored 0–2 were classified as food secure, and those who scored 3 or higher were classified as food insecure.

#### Generalized anxiety

To assess generalized anxiety, we used the 7-item Generalized Anxiety Disorder scale [26], which measures the frequency of anxiety-related experiences over the last two weeks, including nervousness, uncontrollable or excessive worry, restlessness, difficulty relaxing, irritability, and fear of impending doom. Items were rated on a 4-point Likert scale from 0 (not at all) to 3 (nearly every day). Total scores were computed by summing all seven items. Data were then dichotomized as follows: no or mild anxiety for participant scores < 10 and moderate/severe anxiety for participants scoring ≥ 10 [27].

#### Depressive symptoms

To assess depressive symptoms, the Center for Epidemiologic Studies Depression Scale, 10-item Boston form (CES-D-10) was used [28]. This scale asked participants how often in the past week they experienced symptoms such as feeling depressed, having trouble concentrating, or feeling lonely. Each item was rated on a 4-point Likert scale from 0 (rarely or none of the time) to 3 (almost all the time). Participants were asked how often they felt this way during the past week. Scores were calculated as the sum of all item responses, ranging from 0 to 30, with higher scores indicating greater depressive symptom severity. According to established cutoff values, summed totals were subsequently dichotomized as no or minimal depressive symptoms for participants who scored < 10 and moderate/severe depressive symptoms for participants who scored ≥ 10 [28].

#### Adolescent stress

The abbreviated version of the Adolescent Stress Questionnaire (ASQ-S) for adolescents, consisting of 27 items, was used to measure adolescent stress [29]. The ASQ-S assessed a range of stressors, including home life, academic performance, school attendance, romantic relationships, peer pressure, interactions with teachers, and financial strain, among others. Items were scored on a 5-point Likert scale ranging from 1 (not stressful at all) to 5 (very stressful). Scores were calculated as the sum of all item responses, resulting in possible scores ranging from 27 to 135, with higher scores indicating greater adolescent stress [22].

#### Adolescent aggression

Adolescent aggression was assessed using a 17-item Youth Self-Report (YSR) subscale that examines behaviors and moods associated with external and internal aggression [30, 31]. All YSR items were rated on a 3 point scale, ranging from 1 (not true) to 3 (very/often true). Example items include “I get in many fights,” “I physically attack people,” and “I argue a lot.” Summed scores were computed for the 17 items, resulting in possible scores ranging from 17 to 51, with higher scores indicating increased adolescent aggression.

### Statistical analysis

A combination of descriptive and inferential statistical techniques was used to examine the associations between food insecurity and adolescent psychosocial outcomes. For each outcome variable, we first conducted a bivariate analysis (e.g., t-tests and logistic regressions) to assess unadjusted associations with food insecurity. We then estimated multivariable linear or logistic regression models, depending on the distribution and nature of the outcome, to account for key covariates, including site, gender, nativity, socioeconomic status (proxy measure, estimated as persons per bedroom) [32], and age. Generalized anxiety and depressive symptoms were analyzed as binary outcomes because both measures have established cutoff values that are commonly used to identify clinically elevated levels of symptoms. Specifically, GAD-7 scores were dichotomized to distinguish no to mild anxiety from moderate to severe anxiety, and CES-D-10 scores were dichotomized to distinguish no depressive symptoms from significant depressive symptoms. Although analyzing these measures continuously would preserve greater variation in symptom severity, the binary approach was selected to enhance interpretability and align our analyses with clinically relevant symptom thresholds. An interaction term between food insecurity and site was tested to evaluate potential site-specific effects, and model fit was compared using likelihood ratio tests. Variables in all models were assessed for multicollinearity using the variance inflation factor (VIFs), with no evidence of problematic collinearity (all VIF values < 2). All analyses were conducted in RStudio (version 2024.12.0 + 467), with a significance level of *p* < .05. Because these analyses were descriptive and exploratory, no corrections for multiple testing were applied.

## Results

### Sample characteristics and site comparisons

The analytic sample included 300 HL adolescents from Los Angeles (*N* = 195) and Miami-Dade (*N* = 105) counties. The mean age of participants was 15.3 years (SD = 0.74), with a median age of 15.0 years. A majority of participants identified as female (60.7%). Most participants were in 10th grade (50.7%), followed by 11th grade (30.0%) and 9th grade (19.3%). Overall, 80.0% of participants were born in the United States, with a substantial site-level difference: 94.4% of adolescents in Los Angeles were U.S.-born compared to 53.3% in Miami-Dade (*p* < .001). Regarding parental nativity, 79.0% of mothers and 82.7% of fathers were born outside of the United States. In terms of the highest parental education level, 31.3% of participants reported that their parents did not graduate from high school, while only 6.0% reported having a parent with a postgraduate degree (Table 1).

**Table 1:** Analytic sample characteristics and site comparisons

	Los Angeles (*N* = 195)	Miami (*N* = 105)	Overall (*N* = 300)
**Age**			
14	33 (17%)	10 (9.6%)	43 (14.3%)
15	88 (46%)	47 (45%)	135 (45%)
16	67 (35%)	41 (39%)	108 (36%)
17	5 (2.6%)	6 (5.8%)	14 (4.7%)
Mean (SD)	15.2 (0.76)	15.4 (0.75)	15.3 (0.76)
Median [Min, Max]	15.0 [14.0, 17.0]	15.0 [14.0, 17.0]	15.0 [14.0, 17.0]
**Grade Level**			
Freshman	30 (15.4%)	28 (26.7%)	58 (19.3%)
Sophomore	95 (48.7%)	57 (54.3%)	152 (50.7%)
Junior	70 (35.9%)	20 (19.0%)	90 (30.0%)
**Gender**			
Male	75 (38.5%)	43 (41.0%)	118 (39.3%)
Female	120 (61.5%)	62 (59.0%)	182 (60.7%)
**Socioeconomic Status (Persons per Bedroom)**			
Mean (SD)	2.59 (1.17)	1.65 (0.61)	2.19 (1.05)
**Nativity**			
Born in the United States	184 (94.4%)	56 (53.3%)	240 (80.0%)
Not Born in the United States	11 (5.6%)	49 (46.7%)	60 (20.0%)
**Mother’s Birthplace**			
Born in the United States	49 (25.1%)	13 (12.4%)	62 (20.7%)
Not Born in the United States	146 (74.9%)	91 (86.7%)	237 (79.0%)
**Father’s Birthplace**			
Born in the United States	36 (18.5%)	14 (13.3%)	50 (16.7%)
Not Born in the United States	158 (81.0%)	90 (85.7%)	248 (82.7%)
**Highest Parental Education Level**			
Did not graduate high school	85 (43.6%)	9 (8.6%)	94 (31.3%)
High school graduate	45 (23.1%)	21 (20.0%)	66 (22.0%)
Some college	45 (23.1%)	24 (22.9%)	69 (23.0%)
College graduate	14 (7.2%)	37 (35.2%)	51 (17.0%)
Postgraduate degree	4 (2.1%)	14 (13.3%)	18 (6.0%)
**Food Security Status**			
Food Secure	151 (77.0%)	91 (87.0%)	242 (80.7%)
Food Insecure	44 (23.0%)	14 (13.0%)	58 (19.3%)
**Anxiety Severity**			
No/Mild Anxiety	144 (74.0%)	85 (81.0%)	229 (76.3%)
Moderate/Severe Anxiety	51 (26.0%)	20 (19.0%)	71 (23.6%)
**Depression Classification**			
No depressive symptoms	114 (58.0%)	71 (68.0%)	185 (61.7%)
Significant depressive symptoms	81 (42.0%)	34 (32.0%)	115 (38.3%)

*Totals may not equal 100% owing to rounding errors

Participant characteristics and site comparisons for Hispanic/Latino adolescents in Los Angeles and Miami-Dade (*N* = 300). Values are mean (SD) or n (%). Food insecurity was measured using the 9-item USDA Youth Module. Socioeconomic status was proxied by household crowding, defined as persons per bedroom. Group differences were tested with chi-squared and t-tests

Food insecurity was more prevalent in Los Angeles than in Miami. In Los Angeles, 23% of youth (*n* = 44) were classified as food insecure compared to 13% (*n* = 14) in Miami. This a difference did not reach statistical significance, however ($\:\chi\:$² = 3.16, *p* =.075). Household crowding, measured as persons per bedroom, was significantly higher among participants in Los Angeles *(M = 2.59*,* SD = 1.17)* compared with participants in Miami-Dade *(M = 1.54*,* SD = 0.61; p < .001)*. No statistically significant site differences were observed for gender (*p* = .80), age distribution (*p* =.20), moderate/severe anxiety (*p* = .22), or clinically elevated depressive symptoms (*p* = .15) (Table 1). Taken together, site differences were most apparent for nativity and household crowding, whereas differences in food insecurity and internalizing outcomes did not reach statistical significance.

### Clinically elevated levels of mental health problems

Overall, 23.6% of adolescents met criteria for moderate/severe anxiety symptoms, and 38.3% met criteria for clinically elevated depressive symptoms. Clinically elevated depressive symptoms were more common than moderate/severe anxiety symptoms in the analytic sample. These estimates indicate a substantial burden for internalizing symptoms in the analytic sample, particularly for depressive symptoms. Mean stress and aggression scores were 80.07 (SD = 26.90) and 20.51 (SD = 8.21), respectively. Internal consistency ranged from acceptable to excellent across measures, with Cronbach’s alpha values of 0.75 for the food insecurity scale, 0.92 for the GAD-7, 0.75 for the CES-D-10 measure, 0.94 for the ASQ-S measure, and 0.86 for the YSR aggression subscale.

### Generalized anxiety

Food insecurity was significantly associated with increased odds of moderate to severe generalized anxiety symptoms among HL adolescents. In the unadjusted model, food-insecure youth had over four times greater odds of clinically elevated anxiety compared to their food-secure peers (OR = 4.60, 95% CI: 2.51–8.51, *p* < .001) (Table 2). This association remained significant in the adjusted logistic regression model that included site, gender, nativity, socioeconomic status, and age (OR = 5.39, 95% CI: 2.82–10.45, *p* < .001). In the adjusted model, boys had lower odds of clinically elevated anxiety compared to girls (OR = 0.46, 95% CI: 0.24–0.85, *p* = .015) (Table 2). An interaction term between food insecurity and site was not significant (*p* = .29) and did not improve model fit.

**Table 2:** Logistic & linear regression models predicting anxiety, depressive symptoms, stress, and aggression.

Predictor	Estimated OR	95% CI
**Bivariate Model (Generalized Anxiety)**		
Food Insecure (ref = Food Secure)	4.60	2.51–8.51
**Multivariable Model (Generalized Anxiety)**		
Food Insecure	5.39	2.82–10.45
Site (ref = Los Angeles)	1.03	0.47–2.21
Gender (ref = Female)	0.46	0.24–0.85
Nativity (ref = U.S.-born)	0.83	0.34–1.94
Socioeconomic Status (Persons per Bedroom)	1.27	0.97–1.67
Age	1.30	0.88–1.94
**Bivariate Model (Depressive Symptoms)**		
Food Insecure (ref = Food Secure)	3.17	1.77–5.77
**Multivariable Model (Depressive Symptoms)**		
Food Insecure	3.13	1.72–5.81
Site (ref = Los Angeles)	0.83	0.43–1.58
Gender (ref = Female)	0.54	0.32–0.9
Nativity (ref = U.S.-born)	0.89	0.43–1.83
Socioeconomic Status (Persons per Bedroom)	1.05	0.82–1.34
Age	1.06	0.76–1.47
Predictor	Estimated Difference in Mean	95% CI
**Multivariable Model (Adolescent Stress)**		
Food Insecure	0.39	0.12–0.65
Site (ref = Los Angeles)	−0.18	−0.46–0.09
Gender (ref = Female)	−0.55	−0.77 - −0.34
Nativity (ref = U.S.-born)	−0.15	−0.45–0.16
Socioeconomic Status (Persons per Bedroom)	−0.04	−0.14–0.07
Age	−0.04	−0.18–0.1
**Multivariable Model (Adolescent Aggression)**		
Food Insecure	0.25	−0.03–0.53
Site (ref = Los Angeles)	−0.41	−0.7 - −0.12
Gender (ref = Female)	−0.18	−0.41–0.05
Nativity (ref = U.S.-born)	0.08	−0.24–0.4
Socioeconomic Status (Persons per Bedroom)	−0.03	−0.14–0.08
Age	−0.07	−0.22–0.08

Abbreviations: OR = odds ratio, CI = Confidence Interval

Associations between household food insecurity and adolescent psychosocial outcomes. Logistic models estimate odds of moderate to severe anxiety (GAD-7) and significant depressive symptoms (CESD-10). Linear models estimated the differences in standardized scores for stress (ASQ-S) and aggression (YSR-Aggression). Results show as OR (95% CI) for logistic models and difference in means (95% CI) for linear models

### Depressive symptoms

Food insecurity was significantly associated with clinically elevated depressive symptoms. In the unadjusted model, food-insecure adolescents had significantly higher odds of clinically elevated depressive symptoms compared with food-secure adolescents (OR = 3.17, 95% CI: 1.77–5.77, *p* < .001) (Table 2). This association remained statistically significant after adjusting for covariates (OR = 3.13, 95% CI: 1.72–5.81, *p* < .001). We also found that boys were less likely than girls to report clinically elevated depressive symptoms than girls (OR = 0.54, 95% CI: 0.32–0.90, *p* = .020) (Table 2). A marginal site interaction was detected (Likelihood Ratio Test $\:\chi\:$² = 3.78, *p* = .059), with stratified models indicating a stronger association in Los Angeles.

### Adolescent stress

In the unadjusted linear regression model, food insecurity was significantly associated with higher stress scores, with food-insecure adolescents reporting stress levels that were, on average, 0.45 standard deviations higher than their food-secure peers (Estimated difference in means = 0.45, 95% CI: 0.17–0.74, *p* = .002). This association remained significant in the adjusted model. Specifically, adolescents who were food insecure had stress scores that were, on average, 0.39 standard deviations higher than their food-secure peers (Estimated difference in means = 0.39, 95% CI: 0.12–0.65, *p* =.005), even after adjusting for site, gender, nativity, socioeconomic status, and age. Gender also emerged as a significant predictor of adolescent stress, with male participants reporting significantly lower stress scores than girls (Estimated difference in means = −0.55, 95% CI: −0.77 to −0.34, *p* < .001) (Table 2). No statistically significant associations were observed for site, nativity, SES, or age. A food insecurity × site interaction was tested to examine whether the association varied between Los Angeles and Miami, but was not statistically significant (*F* = 1.28, *p* = .259).

### Adolescent aggression

In the unadjusted model, food insecurity was significantly associated with higher adolescent aggression scores (Estimated difference in means = 0.32, 95% CI: 0.04–0.53, *p* = .027), suggesting a positive bivariate relationship before adjusting for covariates. However, this association became nonsignificant after adjusting for covariates. Among covariates, site emerged as a significant predictor of aggression. Adolescents in Miami reported significantly lower aggression scores compared with those in Los Angeles (Estimated difference in means = −0.41, 95% CI: −0.70 to −0.12, *p* = .006). No other covariates, including gender, nativity, SES, or age, were significantly associated with aggression.

## Discussion

We found that food insecurity was most consistently associated with internalizing and stress-related outcomes among HL adolescents in Los Angeles and Miami. Adolescents experiencing food insecurity had greater odds of moderate to severe anxiety and clinically elevated depressive symptoms, as well as higher levels of stress, whereas the adjusted association with aggression was not statistically significant. This pattern suggests that food insecurity may be more closely tied to internalizing than to externalizing behavior in this sample. Although the cross-sectional design we used prevents causal inference, our findings are consistent with prior research linking food insecurity to psychosocial distress and adverse mental health outcomes among adolescents and other vulnerable populations [20, 33]. Taken together, these results reinforce the relevance of food insecurity as a correlate of psychosocial vulnerability among HL adolescents, particularly for internalizing symptoms and stress.

The composition of the sample also warrants consideration. Although most adolescents were U.S.-born, the majority had foreign-born parents, reflecting a largely second-generation population embedded in immigrant-family contexts. This is important because food insecurity among adolescents may reflect not only individual experiences of food-related hardship, but also broader family circumstances related to economic strain, household crowding, parental education, language barriers, and navigation of social services. The inclusion of adolescents from Los Angeles and Miami also captures HL youth from two large urban regions with distinct migration histories and predominant HL subgroups, providing an opportunity to examine food insecurity and psychosocial health in a sample that is both demographically specific and contextually heterogenous.

Site and gender differences also emerged across outcomes. Girls reported significantly higher levels of stress, anxiety, and depressive symptoms compared to boys, which is consistent with broader developmental and epidemiological research documenting higher internalizing symptom burden among adolescent girls [34–37]. Site differences were observed for aggression, with adolescents in Los Angeles reporting higher aggression scores than adolescents in Miami. However, the present study did not measure contextual factors that could explain this difference, such as school climate, neighborhood conditions, or exposures to community stressors, limiting our ability to identify factors that may account for this site difference.

The food insecurity by site interaction terms were not statistically significant suggesting that the associations of food insecurity with anxiety, depressive symptoms, and stress did not meaningfully differ between the Los Angeles and Miami sub-samples. This finding is notable given prior research documenting meaningful differences between Los Angeles and Miami HL adolescent samples, including differences in predominant heritage subgroups, migration histories, perceived context of reception, and politically based cultural stressors [22, 23, 38, 39]. These site differences also reflect broader immigration policy histories that may shape the social and structural contexts in which HL families live. For example, Cuban migrants have historically had nationality-specific pathways to legal permanent residence under the Cuban Adjustment Act and the former wet-foot, dry-foot policy, whereas Mexican and Central American migrants have not had access to comparable group-specific adjustment mechanisms [40, 41]. At the same time, the absence of statistically significant site interactions does not establish that food insecurity operates through identical mechanisms across local contexts. Rather, these findings suggest that food insecurity was consistently associated with internalizing and stress-related outcomes across both urban settings, while leaving open questions about the contextual mechanisms that may shape these associations.

### Limitations

Several limitations associated with this study design and sample should be noted. First, the cross-sectional nature of the study prevents us from drawing conclusions about causality or directionality between food insecurity and mental health outcomes. Second, the study relied on self-reported data, which may have introduced bias, particularly with respect to sensitive topics such as aggressive behavior or food-related hardship. In addition, anxiety and depressive symptoms were assessed using screening instruments rather than clinical diagnostic tools, and dichotomizing these measures may have reduced variability in symptom severity. However, this approach allowed us to examine associations with established thresholds for elevated anxiety and depressive symptom burden. Third, data were collected during the early months of the COVID-19 pandemic, when stay-at-home orders were in effect. These conditions may have heightened food insecurity and psychological distress, potentially inflating estimates and limiting generalizability to non-pandemic contexts. Fourth, the sample was limited to adolescents in Los Angeles and Miami, two major urban centers in the United States and may not accurately represent the experiences of HL youth in rural settings. Lastly, although our models adjusted for household crowding, operationalized as persons per bedroom; this measure is an imperfect proxy for socioeconomic status. Although household overcrowding captures one dimension of household economic strain [19, 32], it may not fully reflect household income, wealth, employment stability, parental education, or other forms of material hardships that may shape food insecurity and adolescent mental health. In addition, although county-level household crowding estimates were used to describe the broader context of Los Angeles and Miami-Dade counties, those estimates may not be directly comparable to the study measure of persons per bedroom. Future studies should incorporate more comprehensive measures of socioeconomic position and material hardship.

## Conclusions

The present examination of HL adolescents in two major metropolitan centers supports the growing body of evidence demonstrating that food insecurity is an critical determinant of mental and behavioral health among this adolescent population. Across Los Angeles and Miami, food insecurity was associated with higher levels of anxiety, depressive symptoms, and stress, even after accounting for sociodemographic factors. Although the association with aggression was not statistically significant, the direction and magnitude of the effect suggest a potential link that merits further investigation. Gender and site-level differences observed in this study highlight the importance of differentiating mental health outcomes within HL populations to better understand how structural barriers and local contextual factors shape adolescent health and well-being.

In conclusion, these findings underscore the need for integrated approaches that address both food insecurity and its associated psychological consequences in HL youth. Although many students may have access to school-based nutrition programs, availability alone may not ensure that food-related needs are identified or adequately addressed. School- and community-based efforts should consider routine screening for food-related hardship, confidential referral pathways to existing nutrition and social support resources, as well as culturally responsive mental health supports for adolescents experiencing anxiety, depressive symptoms, or elevated stress. These efforts may be especially important for HL families navigating economic hardship, language barriers, immigration-related concerns, and limited access to behavioral health care. Future longitudinal research may benefit from considering how food insecurity affects psychosocial health over time and identifying family, cultural, and community-level protective factors that may mitigate the mental health consequences of chronic food-related hardship among HL adolescents.

## Data Availability

Data used in this analysis will be made available upon request.
